# Renal cell carcinoma choroidal metastasis recorded by smartphone with interface eyepiece adapter mounted on slit lamp: A case report

**DOI:** 10.1097/MD.0000000000024910

**Published:** 2021-03-12

**Authors:** Qi-bin Xu, Zhi-yi Hu, Shuang-qing Wu

**Affiliations:** a Department of Ophthalmology, Zhejiang Medicine and Western Medicine Integrated Hospital, Hangzhou Red-Cross Hospital; b The Second Clinical Medical College, Zhejiang Chinese Medical University, Hangzhou, China.

**Keywords:** choroid, eyepiece adapter, metastasis, renal cell carcinoma, slit lamp, smartphone

## Abstract

**Rationale:**

Ocular metastasis of renal cell carcinoma (RCC) is rare, and mainly located on the choroid. We report a choroidal metastasis from RCC, which was recorded by a smartphone with an interface eyepiece adapter mounted on a slit lamp.

**Patient concerns:**

A 45-year-old female presented with 1-month history of painless occlusion of the vision field on the left eye, who had undergone right nephrectomy for RCC 19 months ago.

**Diagnoses:**

A smooth, hemispherical and brown protrusion was found behind the pupil nasally. An enhanced computed tomography scan of the orbit showed a slightly high-density hemispherical nodule involving the nasal portions of the left eyeball, the enhancement of the lesion was obvious and homogeneous. A metastatic choroidal space-occupying lesion from RCC was highly suspected according to the clinical and radiological findings.

**Interventions:**

The patient was advised to undergo further treatment, such as radiotherapy.

**Outcomes:**

The images of choroid metastasis were recorded by the smartphone with the interface eyepiece adapter mounted on the slit lamp handily.

**Conclusions:**

The smartphone with an interface eyepiece adapter mounted on the slit lamp can be widely used to record the precious images in the clinic in a timely manner.

## 1 Introduction

Smartphones have been widely used in the medical field, especially in ophthalmology. Smartphones can be used as a useful ophthalmic device to capture images of the anterior and posterior segments of the eye.^[1]^ Aswin et al^[2]^ used a 2x zoom on Nokia 7 Plus with illumination from a slit lamp to take anterior segment photos. Chan et al^[3]^ designed a slit lamp adaptor assembled on the smartphone to function as an anterior segment camera. Pujari et al^[4]^ used a smartphone clipped with a commercially available magnifying lens to define anterior segment photography. Therefore, we combined a smartphone with an interface eyepiece adapter mounted on a slit lamp to capture photography in clinic.

Metastasis of renal cell carcinoma (RCC) to the eyes is rare.^[5]^ The ocular metastasis of RCC is most likely to involve the choroid, iris and ciliary body. We report a case of choroidal metastases of RCC at 19 months after surgery recorded by the smartphone with an interface eyepiece adapter mounted on the slit lamp.

## 2 Case presentation

**Figure 1:**
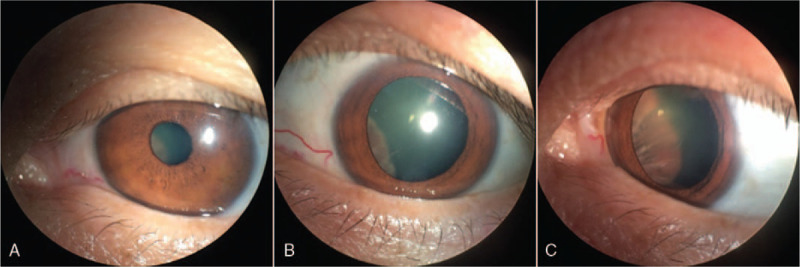
A smooth, hemispherical and brown protrusion involving the nasal portions of the left eyeball which was obvious after mydriasis.

**Figure 2:**
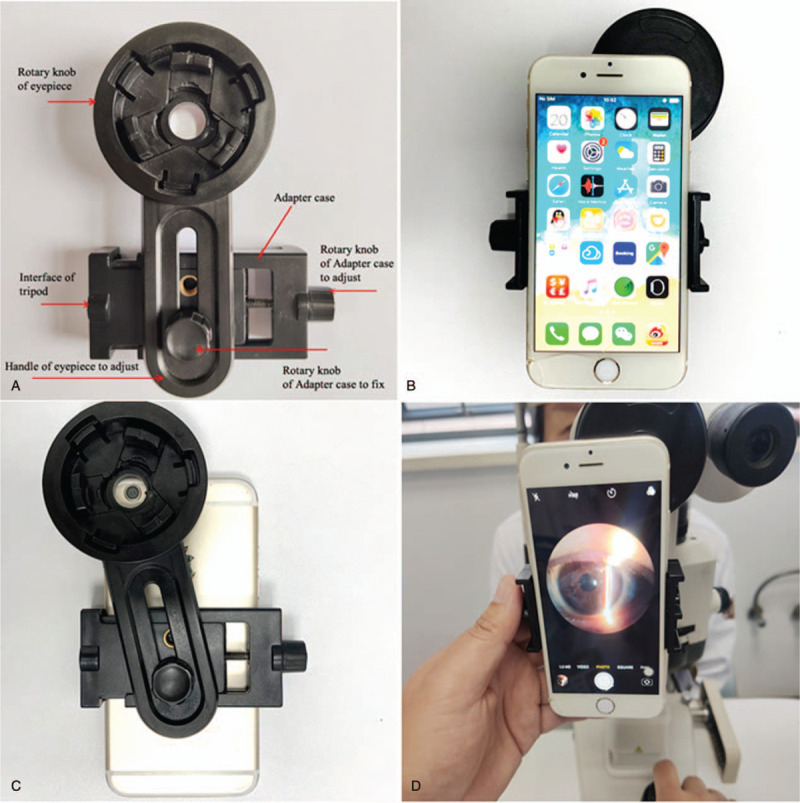
(A) Structure of adapter; (B) A universal eyepiece adapter and a smartphone; (C) A smartphone with an interface eyepiece adapter mounted on a slit lamp to capture anterior segment imaging.

A 45-year-old woman presented with a 1-month history of painless occlusion of the vision field on the left eye, without other eye symptoms. The best corrected visual acuity was 20/25 and intraocular pressure was 18 mm Hg in the left eye. A smooth, hemispherical and brown protrusion on the nasal side behind the pupil was found by the slit lamp microscopy, which was more obvious after mydriasis (Fig. 1). We recorded the images by using a smartphone with an interface eyepiece adapter mounted on the slit lamp. The procedure of image acquisition is presented as follows: Put the smartphone into the adapter case, then adjust the smartphone camera lens aligned with the hole in the back of adapter, and fix the smartphone. Fix the adapter with smartphone on the 1 eyepiece of the slit lamp microscopy by rotating the rotary of eyepiece. Open the smartphone camera and capture the images after focusing (Fig. 2).

The patient had a history of right nephrectomy for RCC 19 months ago, and past medical records showed no involvement of regional lymph nodes. She has not received radiotherapy or chemotherapy after surgery.

**Figure 3:**
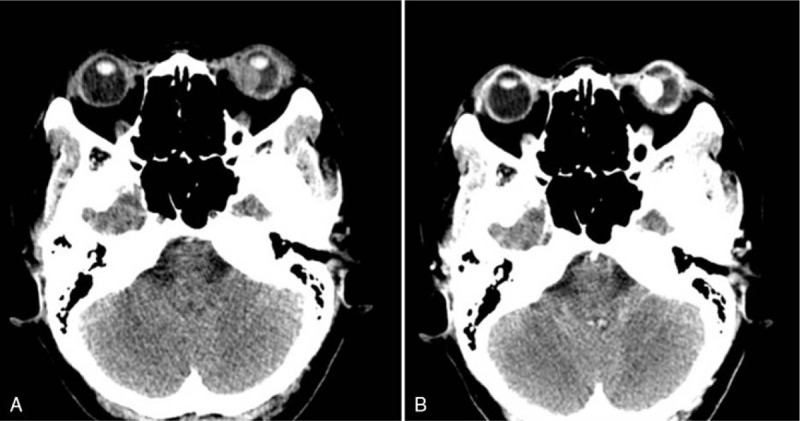
(A) An enhanced computed scan showed a slightly high-density nodule which was found on the nasal side of the left eyeball, with clear boundary and intact eyeball. (B)The enhancement of the lesion was obvious and homogeneous.

An enhanced computed tomography scan of the orbit showed a slightly high-density hemispherical nodule involving the nasal portions of the left eyeball, the enhancement of the lesion was obvious and homogeneous (Fig. 3). Metastatic choroidal space-occupying lesion complicated with retinal detachment was diagnosed. According to the history, clinical and radiological findings, the choroid metastasis of RCC was highly suspected. The results of the renal function test, liver function test and tumor markers were normal. The patient was advised to undergo further treatment, such as radiotherapy, but she refused.

## 3 Discussion

Lung and breast cancers are the most common malignant tumors with ocular metastasis, representing 47% and 21% of metastases, and the choroid is the main location of ocular metastasis, less commonly the ciliary body and iris.^[6]^

The most common sites of metastasis from RCC are the lung (50%) and bone (33%); ocular metastasis of RCC is rare. The ocular metastasis of RCC is most likely to involve choroid in 88%, the ciliary body in 2% and iris in 9%,^[6]^ which may occur at the beginning of the diagnosis of RCC, also have been reported to occur only a few years or even 20 years after nephrectomy.^[3,7]^ Our case showed lesions suggestive of choroidal metastasis at 19 months after nephrectomy.

When the tumor occupation is small, patients may have no symptoms. When it becomes bigger, the most common symptoms are vision loss, blurred vision and occlusion of visual field. After ocular metastases, successful surgical removement of the tumor has been reported,^[8]^ and some authors recommend radiotherapy. Enucleation may be the last consideration for blind eyes.^[9–10]^

Choroidal metastasis of RCC is rarely reported. Preservation of such case data is particularly valuable. Ophthalmology is a subject based on morphology. Collection of ophthalmic data is very important in clinic. It can provide an objective diagnostic basis for clinics and accumulate valuable experience for doctors. Using a smartphone with an interface eyepiece adapter mounted on the slit lamp microscopy can record the clinical manifestation in a timely, accurate, and convenient manner. Conventional slit lamp camera is not portable,^[11]^ and digital slit lamp devices are expensive and cannot be carried easily.^[12]^ Moreover, copying the data from the devices is troublesome. With the popularity of smartphones, ophthalmologists should consider carrying a smartphone to capture ocular imaging as far as possible, so that the acquired data can be stored in smartphones and shared with other ophthalmologists for remote consultation at any time.^[13]^

A 2x zoom on Nokia 7 Plus with illumination from a slit lamp^[2]^ can be used for taking anterior segment photos, but such phone requires a high-resolution camera and it is not cheap. Although the slit lamp adapter designed by Chan ^[3]^ is indeed a method to capture photo of anterior segment, the manufacturing process of the adapter is complicated and it is difficulty to popularize. A smartphone clipped with a commercially available magnifying lens^[4]^ is a diffuse lighting shooting method and only captures a simple panorama of the anterior segment, whereas there is a lack of focusing on details by slit light or capturing images by rear lighting.

We combined a smartphone with an interface eyepiece adapter mounted on a slit lamp microscopy to capture anterior segment images conveniently and economically. Moreover, it can acquire various images by combining with all kinds of slit lamp shooting methods. Our equipment of image acquisition can be easily acquired and widely used. The learning process of collecting photos is also easy, especially for capturing anterior segment imaging.

## 4 Declaration of patient consent

The authors certify that they have obtained all appropriate patient consent forms. In the form, patients have given their consent for their images and other clinical information to be reported in the journal. The patients understand that their names and initials will not be published and due efforts will be made to conceal their identity, but anonymity cannot be guaranteed.

## Author contributions

**Data curation:** Qibin Xu.

**Formal analysis:** Shuangqing Wu.

**Resources:** Qibin Xu.

**Writing – original draft:** Qibin Xu, Zhiyi Hu.

**Writing – review & editing:** Qibin Xu, Shuangqing Wu.
